# Catheter-Related Bloodstream Infection Caused by *Mycolicibacterium iranicum*, California, USA

**DOI:** 10.3201/eid2901.220851

**Published:** 2023-01

**Authors:** Elizabeth L. Ranson, Rebecca K. Tsevat, Benjamin von Bredow, Edwin Kamau, Shangxin Yang, Kavitha K. Prabaker

**Affiliations:** Author affiliations: University of California, Los Angeles, California, USA

**Keywords:** *Mycolicibacterium iranicum*, bacteria, respiratory infections, tuberculosis and other mycobacteria, nontuberculous mycobacterium infections, bacteremia, next-generation sequencing, whole-genome sequencing, California, United States

## Abstract

We describe a case of catheter-related bacteremia caused by *Mycolicibacterium iranicum* in the United States. The case highlights the value of using next-generation sequencing to identify infrequent and emerging pathogens and the challenges associated with choosing appropriate treatments because of limited knowledge of drug resistance mechanisms in those emerging pathogens.

*Mycolicibacterium iranicum* is a rapidly growing mycobacterium (RGM) and emerging cause of respiratory, wound, blood, and central nervous system infections (*1*,*2*). Phylogenetic analyses have shown that *M. iranicum* is more closely related to environmental mycobacterial species than pathogenic species (*3*), and most outbreaks have been associated with exposure to contaminated water (*4*,*5*).

Reports of nontuberculous mycobacteria infections have been increasing worldwide (*6*,*7*), predominantly in immunocompromised patients with hematologic or oncologic medical conditions (*6*). The rise in RGM detection is likely because of increased prevalence of immunocompromising conditions and improved access to molecular diagnostics (*7*). Molecular techniques, especially sequencing multiple conserved genes, such as *rrs* (16S rRNA), *rpoB*, and *groEL* (*hsp65*) (*4*), have led to a dramatic increase in mycobacterial species identified during the past 30 years. We describe a case of *M. iranicum* bacteremia associated with a long-term percutaneous catheter in an immunocompromised patient.

A woman, 76 years of age, with a history of polymyositis and hypertrophic obstructive cardiomyopathy was admitted to an academic hospital in Los Angeles, California, USA, because of substernal chest pain and dyspnea that began 1 day before. Her medications included prednisone (15 mg/d) and intravenous immunoglobulin (20 g administered every 10 days through a port-a-cath that had been in place for several years). The patient had taken mycophenolate mofetil until a month before hospital admission. During each intravenous immunoglobulin infusion over the past 2 years, she had experienced fevers, which were attributed to an infusion reaction. The most recent infusion was 4 days before admission. 

The patient reported fatigue and generalized weakness for several days and an unintentional 25-pound weight loss over the past year. On hospital day 2, she was febrile with a temperature of 101°F (Appendix Figure). Results of a preliminary work-up were unrevealing; however, after 4 days of incubation, multiple aerobic blood cultures (in BACTEC FX aerobic and F lytic media; Becton Dickinson, https://www.bd.com) taken from her port grew beaded, gram-positive rods with yellow mycobacteria-like colonies (Appendix Figure). Matrix-assisted laser desorption/ionization time-of-flight (MALDI-TOF) mass spectrometry failed to identify the isolate. We performed a laboratory-developed, next-generation sequencing-based test that identified the organism as *Mycolicibacterium iranicum*, which we further verified using k-mer–based phylogenic analysis (Figure) (*8*). Using a previously described method for detection of macrolide resistance in *Mycobacteroides abscessus* (*9*), we did not detect a functional *erm* gene.

**Figure:**
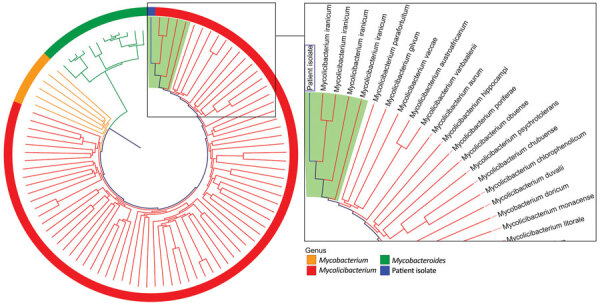
Phylogenetic tree of an isolate from a 76-year old woman in California, USA (blue box), compared with a selection of relevant members of the family Mycobacteriaceae in a case study of catheter-related bloodstream infection caused by *Mycolicibacterium iranicum.* Relatedness was determined by k-mer analysis. Reference genomes were from 4 *Mycolicibacterium iranicum* isolates, 74 other *Mycolicibacterium* spp., 12 *Mycobacteroides abscessus* isolates, and 6 clinically relevant slow-growing *Mycobacterium* spp. Light green background indicates *M. iranicum* isolates. The clinical isolate in this case study was clustered most closely with all 4 *M. iranicum* genomes. Tree is not scale and is designed to show clustering.

When blood cultures demonstrated *Mycobacterium* sp., we changed therapy and administered imipenem, amikacin, and azithromycin. We avoided fluoroquinolones because of the patient’s history of seizures on ciprofloxacin. The patient’s port was extracted. After 13 days, we changed her treatment regimen to doxycycline, azithromycin, and trimethoprim/sulfamethoxazole. Because of intolerable gastrointestinal symptoms, doxycycline treatment was discontinued. The patient was discharged, and azithromycin and trimethoprim/sulfamethoxazole treatments were continued with the presumption that her isolate was macrolide-susceptible because it lacked the *erm* gene. We determined the drug MIC by broth microdilution following Clinical and Laboratory Standards Institute guidelines, and the organism was susceptible to all drugs tested except clarithromycin, to which the organism was resistant with a MIC of 8.0 mg/L. After 4 weeks of therapy, when the MIC results were available, we discontinued azithromycin, added doxycycline, and continued trimethoprim/sulfamethoxazole treatment. The patient again did not tolerate doxycycline. After 6 weeks of targeted therapy, the patient’s fevers resolved, blood cultures were negative, and therapy was ended.

Catheter-related bloodstream infections (CRBSIs) are the most common type of healthcare-associated RGM infection (*6*). Primary risk factors for RGM CRBSIs are immunosuppression, extended catheter placement, and previous antimicrobial therapy; our patient had all 3 risk factors (*4*–*6*). RGM form dense biofilms, which appear to be integral both to their survival in hostile environments and pathogenesis of CRBSIs (*5*,*7*). Successful treatment of nontuberculous mycobacterial CRBSIs usually necessitates catheter removal (*4*,*5*,*10*).

Except for 1 report of bacteremia resistant to clarithromycin, ethambutol, rifabutin, and trimethoprim/sulfamethoxazole, previously reported isolates of *M. iranicum* have been susceptible to all tested drugs (*1*,*2*). Macrolide resistance in RGM is best understood for *M. abscessus* in which specific *rrl* gene mutations mediate constitutive resistance, whereas an intact *erm*(41) gene confers inducible resistance (*9*). We used azithromycin when no *erm*(41)-like gene was detected in the patient’s isolate, but susceptibility testing later revealed macrolide resistance. Macrolide resistance without *erm*(41) or other *erm*-like genes suggests that inducible macrolide resistance may be mediated by a different mechanism. Comparative genomic analysis suggests that *M. iranicum* could acquire multiple drug resistance genes by horizontal transfer (*3*). Molecular resistance mechanisms for *M. iranicum* are not well characterized, and our case highlights the challenges of genotypic resistance prediction in uncommon RGM species.

In summary, we report a case of *M. iranicum* CRBSI that was treated successfully with catheter removal and 2 weeks of amikacin and imipenem followed by 4 weeks of de facto monotherapy with trimethoprim/sulfamethoxazole. This case illustrates the value of using next generation sequencing to identify novel pathogens and the challenges of choosing appropriate treatment because of limited knowledge of drug-resistance mechanisms.

Appendix Additional information for catheter-related bloodstream infection caused by *Mycolicibacterium iranicum*, California, USA.
