# Supplementary material for: Catheter-Related Bloodstream Infection Caused by Mycolicibacterium iranicum, California, USA
Source: Emerg Infect Dis. 2023 Jan;29(1):217–9. doi: 10.3201/eid2901.220851 (PMC9796217; doi:10.3201/eid2901.220851)
Supplement: Appendix — Additional information for catheter-related bloodstream infection caused by Mycolicibacterium iranicum, California, USA. [file 22-0851-Techapp-s1.pdf]

Article DOI: https://doi.org/10.3201/eid2901.220851

Catheter-Related Bloodstream Infection Caused by *Mycolicibacterium iranicum*, California, USA

Appendix

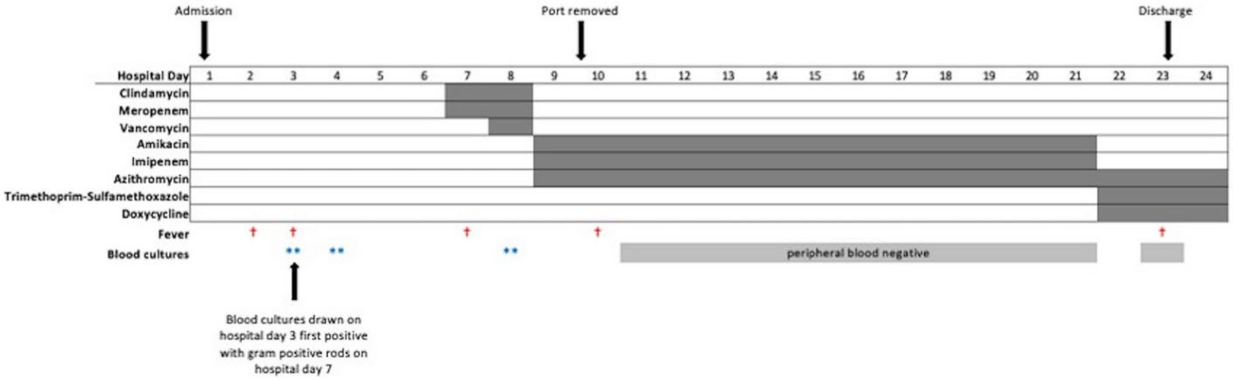

Appendix Figure. Key clinical events and microbiological findings during hospitalization of a patient who had catheter-related bloodstream infection caused by *Mycolicibacterium iranicum* in California, USA. Top row shows the days in the hospital. The top arrows show major clinical events. Antimicrobial drug courses are indicated by gray bars. Red cross (†) indicates fever. Blue asterisks (**) indicate positive blood mycobacterial cultures from the catheter port. No peripheral cultures were drawn on day 22. The patient was discharged on hospital day 23.
